# ChatGPT in Orthopedic Trauma: Consistency, Accuracy, and Agreement With Textbook and Expert Opinion

**DOI:** 10.7759/cureus.110069

**Published:** 2026-06-01

**Authors:** Dror Ronel, Galina Shapiro, Tal Ben Kiki, Yaniv Keren

**Affiliations:** 1 Orthopedic Surgery, Rambam Medical Center, Haifa, ISR

**Keywords:** artificial intelligence in medicine, medical decision-making, proximal femur fracture, subcapital femur fracture, trauma and orthopedic surgery

## Abstract

Background

Artificial intelligence (AI) tools such as ChatGPT are increasingly used in clinical settings, yet their reliability remains unclear.

Methods

This study compared ChatGPT-4o's responses to clinically relevant orthopedic questions on femoral neck and trochanteric fractures with expert consensus and Rockwood and Green's textbook. Seven questions were submitted in repeated sessions to assess consistency. Textual similarity was measured using cosine similarity and Bidirectional Encoder Representations from Transformers (BERT)-based models, alongside readability indices and Cohen's Kappa for agreement.

Results

ChatGPT demonstrated high internal consistency (mean cosine similarity: 0.94-0.98) but only partial agreement with textbook content (mean similarity: 0.88; Cohen's Kappa: 0.16) and expert opinion survey (Cohen's Kappa: 0.16). Discrepancies were noted in age thresholds for arthroplasty and preferred nail length in subtrochanteric fractures, suggesting reliance on outdated sources.

Conclusion

Although ChatGPT provided coherent and readable answers, knowledge gaps persist. This highlights the need for curated, domain-specific models and transparent data sourcing. AI shows promise but should complement, not replace, clinical judgment and up-to-date references.

## Introduction

In recent years, there has been a significant increase in the use of artificial intelligence (AI) technologies, largely driven by the emergence of tools based on large language models (LLMs), such as ChatGPT. The ability to fine-tune these tools for specific tasks has opened a wide range of potential applications. AI tools have demonstrated the capability to pass medical licensing exams [1], including early-stage board exams [2], and - with proper training - even diagnose fractures with high accuracy [3-6]. The ability of AI to solve clinical questions is still debatable, with some studies showing promising results [7,8] and some being limited [9].

In orthopedics, the potential applications of AI are diverse [10], and several reviews have highlighted their use in specific subspecialties, such as spine surgery [11,12] and foot and ankle surgery [13].

Despite their advantages, some AI models do not always perform better than standard regression methods, and most lack external validation [14]. LLMs have the potential to generate inaccurate or misleading responses [15]. It is important to remember that AI is not always consistent, and hallucinations are a critical safety issue [16,17].

Many clinicians and patients increasingly use ChatGPT and similar tools to explore medical questions in daily practice. Previous studies have found that the responses generated by such models can be useful for patient education and for explaining medical conditions such as total hip arthroplasty [18], scoliosis [19], and Kienböck's disease [20].

In their systematic review of the role of LLMs in orthopedic surgery, Zhang et al. found that even though these models have many promising applications but may often generate inaccurate and overcomplex answers [21]. One study compared board-certified orthopedic surgeons to ChatGPT in common emergency department scenarios and found that AI responses were non-inferior and often superior [22]. In contrast, other publications have raised concerns about LLM's ability to predict appropriate treatment in patients with osteoarthritis [23] and hip fractures [24].

Evidence-based medicine presents ongoing challenges. The growing amount of medical knowledge and evidence is not easily translated into practice, especially in surgical disciplines [25]. Clinical complexity, limited sample sizes, and variation in outcomes make it difficult to create robust evidence that supports all clinical questions [25]. Not all evidence is translated to medical practice [26,27], and even guidelines and recommendations are not always followed [28-30].

Proximal femur fractures are a common cause for hospitalization in the elderly and are associated with increased morbidity and mortality in the elderly [31-33]. Its globally increasing incidence presents major public health concerns [34]. Management is complex and necessitates balancing patient and fracture factors in order to get the best outcomes [32]. Proximal femur fractures have become one of the most studied areas of AI implications in orthopedics [14].

To the best of our knowledge, a few studies [12,22-24] have evaluated the reliability of LLM-generated responses to clinical questions posed by physicians. Some used expert opinion, and some used guidelines for the comparison. The aim of this study was to evaluate the consistency, readability, and agreement of ChatGPT-generated responses to a predefined set of seven clinically relevant orthopedic trauma questions derived from Rockwood and Green's Fractures in Adults [35] and to compare these responses with textbook recommendations and expert opinion.

## Materials and methods

The chapters "Femoral Neck Fractures" and "Trochanteric Hip Fracture" from Rockwood and Green's Fractures in Adults (Rockwood), 10th Edition [35], were reviewed. Seven clinically relevant questions were defined based on these chapters, each with a clear and concise answer. The questions were selected by the senior author (board-certified orthopedic surgeon) based on topics explicitly addressed in the selected chapters and considered to have clear, guideline-supported answers. Questions were chosen to represent common clinical decision points (e.g., fixation vs arthroplasty, timing of surgery, implant selection). No formal consensus process or independent validation was performed, which may introduce selection bias.

Each question was presented to ChatGPT-4o in a zero-shot prompt, initiating a new, temporary chat session to minimize potential bias from prior interactions or memory. To assess reproducibility, the same questions were submitted three times at one-week intervals (6.4.2025, 13.4.2025, and 22.4.2025).

To compare ChatGPT and textbook responses with expert opinion, the same questions were distributed to orthopedic trauma specialists in Israel. ChatGPT and textbook answers were then coded according to the predefined response options in the questionnaire to enable direct comparison and agreement analysis. This coding was performed in a binary, decision-based manner, aligning each response with one of two clinically relevant options (e.g., partial versus total hip arthroplasty, or sliding hip screw versus cephalomedullary nail).

In order to quantify similarity between the texts, we used the cosine similarity score [36], calculated via a Bidirectional Encoder Representations from Transformers (BERT) model. All three ChatGPT-generated answers for each question were evaluated for internal consistency using cosine similarity. Further readability analysis was done using the Flesch Reading Ease Score and Flesch-Kincaid Grade Level [20].

To complement the cosine-based similarity, ChatGPT was also asked to blindly compare the textbook and AI-generated answers, assigning a meaning-based similarity score between 0 and 1 (where 0 represents total disagreement and 1 represents total agreement).

Different groups answering the survey were compared with chi-square and Fisher's exact tests when applicable, and a Bonferroni correction was added due to multiple comparisons.

Overall agreement between the textbook and ChatGPT was assessed using Cohen's Kappa. Agreement with expert opinion was assessed using paired Cohen's Kappa with the McNemar test to assess directional bias in disagreement. Krippendorff's alpha and Conger's kappa were calculated to measure the agreement between all three modalities altogether.

All statistical analyses were performed in R (version 4.4.3; R Development Core Team, Vienna, Austria). Cosine similarity was calculated using a BERT model in Visual Studio Code (Microsoft® Corp., Redmond, WA), and the meaning similarity score was generated using ChatGPT-4o (OpenAI, San Francisco, CA). Flesch reading ease score was calculated using a free online tool from the website of Good Calculators.

The study was exempted from institutional ethics committee approval because it did not involve any patients.

## Results

Three sets of questions (Table 1) were generated in order to check for reproducibility. Mean cosine similarity between all sets of answers (SD) for question 1 was 0.94 (0.01), question 2 at 0.97 (0.02), question 3 at 0.97 (0.018), question 4 at 0.94 (0.05), question 5 at 0.98 (0.01), question 6 at 0.98 (0.01), and question 7 at 0.88 (0.05). A GPT similarity, based on the meaning of the answers, scored 1 for all, except question 2, which scored 0.9.

**Table 1:** Clinical questions with answers from Rockwood and Green's Fractures in Adults and ChatGPT. The table presents the clinical questions used for comparison, along with the respective answers from the textbook [35] and ChatGPT.

Question	Answer A (Rockwood)	Answer B (ChatGPT)
1. Treatment for healthy 50-year-old with displaced subcapital fracture.	Total hip replacement recommended for >40 y/o mobile patients.	Internal fixation with screws/DHS to preserve femoral head.
2. Treatment for 40-year-old with comorbidities and displaced subcapital fracture.	Arthroplasty considered in younger patients with risk factors.	Hemiarthroplasty recommended for early mobilization.
3. Partial vs total hip arthroplasty for healthy 80-year-old.	Total hip arthroplasty recommended.	Total hip arthroplasty recommended with functional benefits.
4. Timing of surgery for trochanteric hip fracture.	Operate as soon as optimized, preferably <24h.	Operate within 24–48h to reduce complications.
5. Fixation method for stable trochanteric fracture (OTA/AO A1).	Sliding hip screw recommended.	Sliding hip screw (SHS) preferred.
6. Fixation method for unstable trochanteric fracture (OTA/AO A2/3).	Cephalomedullary nail preferred.	Intramedullary nail preferred.
7. Nail length for fracture extending 2 cm below trochanteric region.	Short nail recommended (if <3 cm distal extension).	Long nail preferred for distal support.

Readability was assessed using the Flesch Reading Ease and Flesch-Kincaid Grade Level metrics [20] across three answer sources. The Flesch Reading Ease mean score was 36.3 (SD=10.5), 29.5 (SD= 17.2), and 38.4 (14.7) for the different sets of prompts 1-3, respectively. The Flesch-Kincaid mean grade was 15.4 (SD=3.7), 15.6 (SD=3.8), and 15.7 (SD=2.8) for sets 1-3, respectively. All scores indicate complicated texts at the level of college graduates.

The first set of answers generated by ChatGPT was compared to the textbook. Cosine similarity scores (Table 2) ranged from 0.75 in question 1 to 0.966 in question 6. Mean cosine similarity score was 0.88 with a standard deviation of 0.07. Most questions showed high semantic similarity, though question 1 had notably lower similarity compared to others, showing different phrasing and word content.

**Table 2:** Cosine and GPT similarity scores comparing the first set of answers generated by ChatGPT to Rockwood and Green's Fractures in Adults. Cosine similarity reflects textual similarity, while GPT similarity represents meaning-based agreement (0-1 scale).

Question	Cosine Similarity Score	GPT Similarity Score
1	0.7529	0
2	0.906	0.9
3	0.8881	1
4	0.846	0.8
5	0.9536	1
6	0.9663	1
7	0.8327	0

GPT similarity scores (Table 2) were 1 for questions 3, 5, and 6; 0.9 for question 2; 0.8 for question 4; and 0 for questions 1 and 7.

An expert opinion survey, including answers from board-certified orthopedic surgeons, received 57 answers. Among them, the majority had more than 10 years of experience (38, 72.7%). There were 15 (26.3%) trauma specialists, and 21 (36.8%) specialists from tertiary hospitals. All comparisons between the subgroups showed only slight differences; none of them was statistically significant. Note that, in some questions, the majority of responders to a question was 50%-60%.

Comparison of ChatGPT and Rockwood (Table 3) after coding the answers into the survey's possible answers showed 57% of agreement, with 0.16 Cohen's Kappa (p=0.66, CI: -0.52 to 0.72). ChatGPT had strong agreement with the majority of the voters in each question of the expert opinion survey, with 100% of agreement and Cohen's Kappa of 1 (p=0.008). The comparison between Rockwood and the expert opinion survey resulted in 57% of agreement, with 0.16 Cohen's Kappa (p=0.66, CI: -0.52 to 0.72). McNemar's test showed no evidence of systematic directional disagreement between Survey and Rockwood (p=1.0) or between Rockwood and ChatGPT (p=1.0). The survey and ChatGPT had perfect agreement, so McNemar's test could not be applied. Regarding overall agreement, Krippendorff's alpha was 0.44 (CI: -0.18 to 0.81), and Cohen's Kappa was 0.43 (p=0.15, CI: -0.21 to 1). 

**Table 3:** Paired comparison between expert opinion survey, ChatGPT, and Rockwood and Green's Fractures in Adults. Agreement is presented as percentage agreement, Cohen's kappa, and the McNemar test, where applicable.

Comparison	Percent of Agreement	Cohen’s Kappa	McNemar p
Rockwood vs ChatGPT	57% (4/7)	0.16 (p=0.66, CI: -0.52 to 0.72)	1
Rockwood vs survey	57% (4/7)	0.16 (p=0.66)	1
ChatGPT vs survey	100%	1 (p=0.008, CI: -0.52 to 0.72)	N/A

## Discussion

AI has the potential to synthesize large volumes of clinical data, but its application in decision-making remains limited by data quality and task specificity.

One of the main concerns regarding generative AI is its "black box" nature. The lack of transparency in how an answer is generated raises questions about both accuracy and reproducibility. Another challenge in evaluating generative AI is the potential for bias introduced by memory and user interaction learning. This study employed two strategies to mitigate these concerns: each prompt was entered in a new, temporary chat session to eliminate memory effects, and all prompts were repeated at weekly intervals. All answers remained virtually identical, with only minor differences in phrasing.

Despite recent advances, a knowledge gap persists between the literature and clinical practice [25,26]. Even guidelines and protocols are not always consistently applied in real-world settings [28-30]. Rockwood and Green's Fractures in Adults [35] is considered the gold standard in orthopedic trauma and bases its recommendation on the most recent literature combined with author's experience. Therefore, it was chosen as a benchmark to assess the accuracy of ChatGPT's responses.

To complement this comparison, we conducted a survey among practicing senior orthopedic surgeons. Interestingly, ChatGPT's responses often paralleled the distribution of expert opinions, not only aligning with Rockwood in many areas but also echoing the variability observed in clinical practice. This suggests that, while ChatGPT may lag behind the most updated textbook editions, its outputs reflect prevailing expert consensus and highlight the same areas of ongoing debate among clinicians.

**Figure 1:**
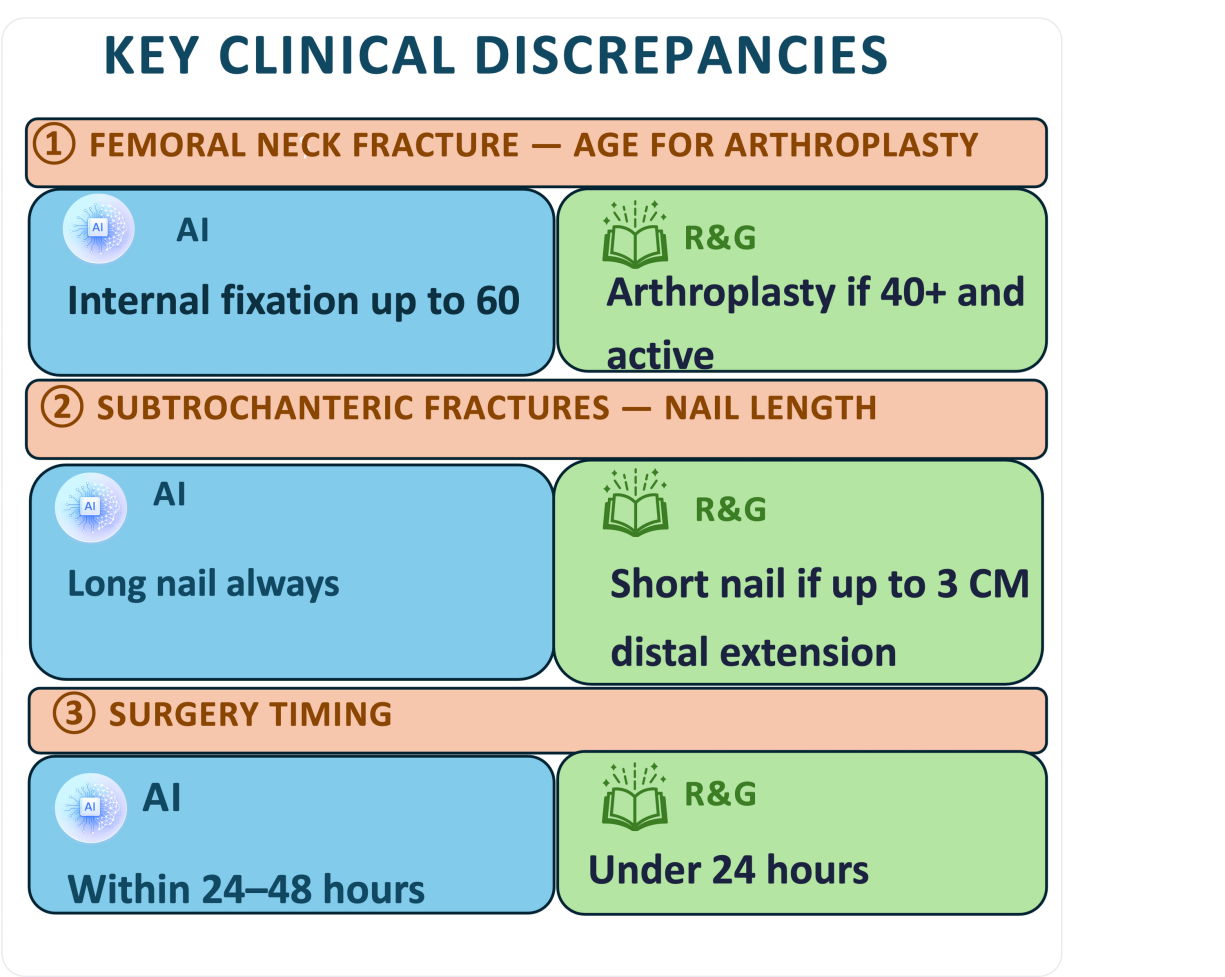
Key discrepancies between AI-generated recommendations and Rockwood and Green's Fractures in Adults. The figure highlights areas of disagreement, including treatment thresholds for femoral neck fractures, timing of surgery, and nail length selection in subtrochanteric extension. R&G: Rockwood and Green's. Image created using PowerPoint (Microsoft® Corp., Redmond, WA) Source: Ref [35]

This study found only partial agreement between ChatGPT and Rockwood. While many questions yielded similar answers, there were a few areas of notable discrepancy (Figure 1).

One such discrepancy was the treatment of subcapital fractures in the younger patients. These fractures are associated with poor outcomes [37,38], and in recent years, a shift has occurred toward recommending arthroplasty in younger age groups [39-41]. The latest edition of Rockwood (2025) [35] recommends arthroplasty for patients over 40, whereas the previous edition (2019) [42] set the threshold at 60 years. ChatGPT's response was aligned with the earlier recommendation.

The optimal time for surgical intervention remains another area of debate. Some authors suggest that mortality increases if surgery is delayed beyond 24 hours [43], while a randomized controlled trial found no significant difference between rapid (up to six hours) or standard care [44]. While the recent version of Rockwood recommends surgery within 24 hours of admission if possible, ChatGPT recommends surgery within 24-48 hours.

Establishing nail length in patients with subtrochanteric extension is another area of controversy. One randomized controlled trial found it safe to use a short cephalomedullary nail (CMN) if the subtrochanteric extension is up to 3 cm [45]. Some argue that a long nail is more stable [46], while others put the emphasis on the distance between the fracture line and the distal locking screw [47]. The current edition of Rockwood recommends the use of a short nail if the subtrochanteric extension is up to 3 cm, while the previous edition treated them as subtrochanteric fractures, recommending long CMN. ChatGPT recommended the use of a long nail in these, aligning with the older approach.

Within the scope of the selected questions, the findings suggest discrepancies between ChatGPT outputs and textbook recommendations, pointing towards a potential knowledge gap in ChatGPT's outputs. While updated editions of textbooks integrate both recent evidence and expert consensus, LLMs generate responses based on probabilistic modeling of vast, unverified datasets. Although LLMs generally produce coherent and plausible answers, the underlying mechanism for content generation remains opaque. Consequently, these models may incorporate outdated or low-quality data, particularly when not explicitly instructed to assess source credibility. 

Future directions

A potential solution is the development of domain-specific AI trained on curated, high-quality data, which may improve accuracy but limit generalizability. Balancing accuracy, transparency, and up-to-date evidence remains a key challenge for integrating LLMs into clinical practice. At the same time, LLM use raises ethical and regulatory concerns: clinicians are accountable for their decisions, which must rely on trustworthy, peer-reviewed evidence, and basing decisions on potentially unreliable AI-generated information is problematic.

The future will involve higher incorporation of AI tools in our lives and work. These tools should be improved and trained in a way that ensures continuous updates and transparency in data sources. For example, a system that includes timestamps for its training set or grades the credibility of its sources could improve reliability. OpenAI has recently introduced HealthBench [48] - a benchmark for assessing the reliability of generative AI in medicine. It is an important tool, but it is not enough to replace independent research. The American Food and Drug Authority recently completed a scientific review pilot using generative AI and plans to expand its use [49].

Future research should focus on real-world clinical validation, comparing LLM-generated recommendations with actual decisions and outcomes.

Limitations

This study has several limitations. First, it is based on a small number of predefined, text-based questions and does not capture the full spectrum of orthopedic decision-making. Second, the question selection process was not validated by an independent panel, introducing potential selection bias. Third, the analysis does not include radiographic interpretation or real-time clinical decision-making. Fourth, semantic similarity metrics may not fully reflect clinical correctness. Finally, the use of ChatGPT itself to generate meaning-based similarity scores introduces potential circularity.

Despite these limitations, the study's design intentionally mirrors real-world use of LLMs by clinicians, providing a pragmatic and clinically relevant assessment of how AI-generated advice holds up against current standards.

## Conclusions

AI and LLMs are increasingly influencing clinical practice, making it essential to critically evaluate their strengths and limitations to ensure safe and effective patient care. In this study, ChatGPT generated generally coherent responses, though not always aligned with the most up-to-date evidence. Within the study's limited scope, the findings highlight inconsistencies not only in AI outputs but also between established references and clinical practice, reflecting a broader gap between current evidence and its implementation. While AI may provide a useful general framework, it cannot yet be relied upon for precise medical decisions, as it lacks consistently current knowledge and clinical judgment. Clinicians must therefore remain actively involved in evaluating these tools and integrating them cautiously into practice, recognizing that AI is an adjunct rather than a substitute for their own decision-making.
